# Neuroinflammation alters cellular proteostasis by producing endoplasmic reticulum stress, autophagy activation and disrupting ERAD activation

**DOI:** 10.1038/s41598-017-08722-3

**Published:** 2017-08-14

**Authors:** Cristina Pintado, Sandra Macías, Helena Domínguez-Martín, Angélica Castaño, Diego Ruano

**Affiliations:** 1 Departamento de Bioquímica y Biología Molecular, Facultad de Farmacia, Universidad de Sevilla, 41012 Sevilla, Spain; 2 Instituto de Biomedicina de Sevilla (IBiS)-Hospital Universitario Virgen del Rocío/Consejo Superior de Investigaciones Científicas/Universidad de Sevilla, 41013 Sevilla, Spain; 3 Facultad de Ciencias Ambientales y Bioquímica, Avenida Carlos III s/n, 45071 Toledo, Spain

## Abstract

Proteostasis alteration and neuroinflammation are typical features of normal aging. We have previously shown that neuroinflammation alters cellular proteostasis through immunoproteasome induction, leading to a transient decrease of proteasome activity. Here, we further investigated the role of acute lipopolysaccharide (LPS)-induced hippocampal neuroinflammation in cellular proteostasis. In particular, we focused on macroautophagy (hereinafter called autophagy) and endoplasmic reticulum-associated protein degradation (ERAD). We demonstrate that LPS injection induced autophagy activation that was dependent, at least in part, on glycogen synthase kinase (GSK)-3β activity but independent of mammalian target of rapamycin (mTOR) inhibition. Neuroinflammation also produced endoplasmic reticulum (ER) stress leading to canonical unfolded protein response (UPR) activation with a rapid activating transcription factor (ATF) 6α attenuation that resulted in a time-dependent down-regulation of ERAD markers. In this regard, the time-dependent accumulation of unspliced X-box binding protein (XBP) 1, likely because of decreased inositol-requiring enzyme (IRE) 1α-mediated splicing activity, might underlie *in vivo* ATF6α attenuation. Importantly, lactacystin-induced activation of ERAD was abolished in both the acute neuroinflammation model and in aged rats. Therefore, we provide a cellular pathway through which neuroinflammation might sensitize cells to neurodegeneration under stress situations, being relevant in normal aging and other disorders where neuroinflammation is a characteristic feature.

## Introduction

Neuroinflammation and proteostasis disruption are characteristics of normal aging and some age-related neurodegenerative diseases. Cellular proteostasis is mainly sustained by the ubiquitin proteasome system and autophagy, which could act coordinately under specific situations^1^. A consequence of proteostasis alteration is ER-stress. Under this situation, cells activate the UPR, which is mediated by IRE1α, ATF6α and double-stranded RNA-dependent protein kinase-like endoplasmic reticulum kinase (PERK) signalling pathways^2^. An efficient UPR activation is necessary for a proper response to cellular challenges in order to resolve stress situations and preserve cell viability. However, others and we have demonstrated that proper UPR activation is disrupted in aged animals leading to neurodegeneration^3–8^, despite the causes of this effect are not well known.

On the other hand, low-grade chronic inflammation is also a marker for aging^9–12^ and represents a factor rising up cell vulnerability to proteasomal stress^13^. Importantly, neuroinflammation, autophagy and UPR are three interrelated processes, which can influence each other depending on different factors and stressors^14, 15^. In this regard, *in vitro* experiments revealed that LPS treatment induced autophagy cell death in an UPR dependent manner^16^. However, autophagy can be also activated by other mechanisms including m-TOR inhibition or both GSK-3β inhibition or activation depending on the cell type or stressors. Moreover, modulation of the inflammatory response and cytokine production is dependent on toll-like receptor (TLR) activation of XBP1 in both macrophages^17^ and rheumatoid arthritis synoviocytes^18^, and also on the cross-talk between GSK-3β and XBP1^19^. In consequence, a better comprehension of the *in vivo* relationships between neuroinflammation, UPR and autophagy would be useful for understanding how the neuroinflammatory response could be modulated in the context of aging or neurodegenerative diseases. Therefore, we set out to investigate the role of LPS-induced neuroinflammation on UPR and autophagy in rat hippocampus. Our results indicate that LPS injection activated both autophagy and UPR. Autophagy activation was depended, at least in part, on GSK-3β activity but independent of mTOR inhibition. On the other hand, LPS injection produced a canonical but short UPR activation. Both ATF6α and IRE-1α were quickly attenuated, leading to down-regulation of ERAD markers. Importantly, the up-regulation of ERAD markers induced by proteasome inhibition was abolished in both aged rats and LPS-injected young animals. These results suggest that neuroinflammation-induced ERAD inactivation might increase cell vulnerability under proteasomal stress situations.

## Results

### LPS injection induces autophagy activation in rat hippocampus

**Figure 1:**
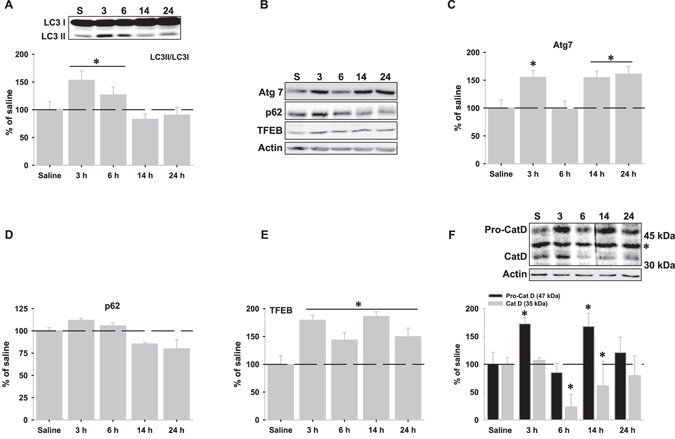
LPS injection induces transient autophagy activation in rat hippocampus. (**A**) Representative western blot of LC3 I and II and graphical representation of quantification of the LC3 II/LC3 I ratio determined by optical density at different times after LPS injection. (**B**) Representative western blots of Atg7, TFEB and p62 at different times after LPS injection. (**C**–**E**) Graphical representation of data from optical density quantification of western blots corresponding to Atg7, TFEB and p62, respectively. (**F**) Representative western blot and graphical representation of quantification by optical density of lysosomal protease cathepsin D at different times after LPS injection. The asterisk indicates a non-specific band. Data are presented as mean ± SD (n = 4) of percentage of variation relative to controls. *p < 0.05, significant differences compared with control (saline-injected) animals.

We have previously demonstrated that LPS-induced neuroinflammation in rat hippocampus led to immunoproteasome induction and transient decrease of proteasome activity^13^. Therefore, we analyzed whether LPS injection could activate autophagy in order to sustain proteolytic activity. As shown, LPS injection induced a rapid and significant increase of some autophagic markers such as light chain 3 (LC3)-II (Fig. 1A) and Atg7 (Fig. 1B,C and Supplementary Fig. S1A). Also, LPS injection triggered specific transcriptional up-regulation of the BCL2 associated athanogene 3 (*bag3*), but not *bag1*, which are involved in delivering proteins for degradation to the autophagy or the proteasome, respectively (Supplementary Fig. S1B and C)^20^. Moreover, the amount of p62 protein, which is considered a marker for autophagic flux, showed a time-dependent decrease, but without statistical significance (Fig. 1B and D). The LPS-dependent increase in autophagic flux was also supported by *in vitro* experiments (Supplementary Fig. S1D). On the other hand, the master transcription factor EB (TFEB), which coordinates both autophagy and lysosomal biogenesis, was significantly increased after LPS injection (Fig. 1B and E) and the expression of the lysosomal marker cathepsin D decreased significantly from 6 to 14 hours, supporting lysosomal degradation of autophagosomes (Fig. 1F). Taken together, these data indicate that LPS injection activates autophagy in rat hippocampus. Importantly, the timeframe of autophagy activation was coincident with the replacement of constitutive proteasomes by the immunoproteasomes (Supplementary Fig. S2).

### LPS-induced autophagy is partially dependent on GSK-3β activity but independent on mTOR inhibition

**Figure 2:**
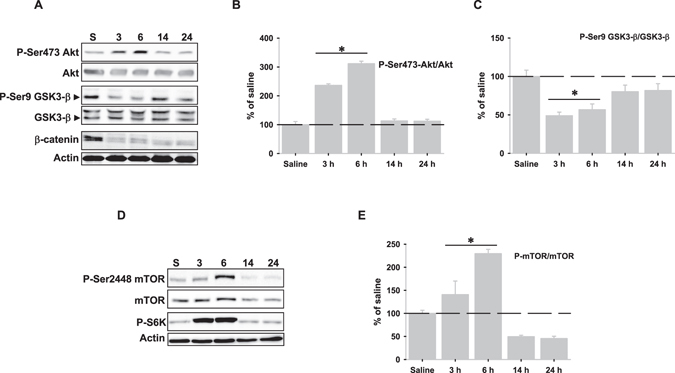
LPS injection activates both Akt/GSK-3β/β-catenin and Akt/mTOR pathways in rat hippocampus. (**A**) Representative western blots of phopho-Ser473-Akt and Akt (upper panel), phospho-Ser9-GSK-3β and GSK-3β (middle panel) and β-catenin (lower panel). (**B**,**C**) Graphical representation of data from optical density quantification of western blots corresponding to phopho-Ser473-Akt/Akt and phospho-Ser9-GSK-3β/GSK-3β ratios, respectively after LPS injection. (**D**) Representative western blots of phopho-Ser2448-mTOR and mTOR (upper panel) and phospho-S6K (lower panel). (**E**) Graphical representation of data from optical density quantification of western blots corresponding to phopho-Ser2448-mTOR/mTOR ratio following LPS injection. Data are presented as mean ± SD (n = 4) of percentage of variation relative to controls. *p < 0.05, significant differences compared with control (saline-injected) animals.

Next, we searched for the cellular pathways that might be involved in LPS-induced autophagy activation. Based on previous data we first focused on the Akt/GSK-3β/βcatenin axis^1, 21^. As shown, LPS injection induced early Akt activation as indicated the higher amount of both phosphoSer473Akt (Fig. 2A upper panel and 2B) and phosphoThr308Akt (data not shown) from 3 to 6 hours. Despite Akt activation, the amount of phosphoSer9-GSK-3β, a target protein of Akt, was significantly reduced during this time, supporting that LPS injection increased basal activity of GSK-3β (Fig. 2A middle panel and 2C). This was confirmed by the significant decrease of β-catenin (Fig. 2A lower panel). We next tested whether GSK-3β activity could be involved in LPS-induced autophagy activation. For that purpose, we performed a new set of experiments using LPS-treated N13 cells in the presence of the GSK-3β inhibitor VII. Similarly as observed *in vivo*, LPS treatment increased the LC3-II/LC3-I ratio. However, it was partially reversed in the presence of the GSK-3β inhibitor VII (Supplementary Fig. S3). These data indicate that LPS-induced autophagy activation is partially dependent on GSK-3β activity.

Because mTOR inhibition is involved in autophagy activation^22^, we also investigated the Akt/mTOR/S6K pathway. As shown in Fig. 2D and E, phosphorylated mTOR at Ser2448 was significantly increased from 3 to 6 hours but decreased at 14 and 24 hours, indicating that mTOR was activated from 3 to 6 hours. This was corroborated by increased phosphorylation of the target proteint of mTOR p70 ribosomal S6 kinase (Fig. 2D lower panel). Taken together, these results show the LPS injection activates both Akt/GSK-3β/βcatenin and Akt/mTOR/S6K pathways, being autophagy activation partially dependent on GSK-3β activity but independent on mTOR inhibition.

### LPS injection induces UPR activation

We next wondered if LPS-induced proteostasis alteration could affect the ER homeostasis. Thus, we searched for ER-stress by evaluating UPR activation. To address this issue, we carried out a detailed molecular analysis of specific markers of the three arms of the UPR.

### IRE1α pathway

**Figure 3:**
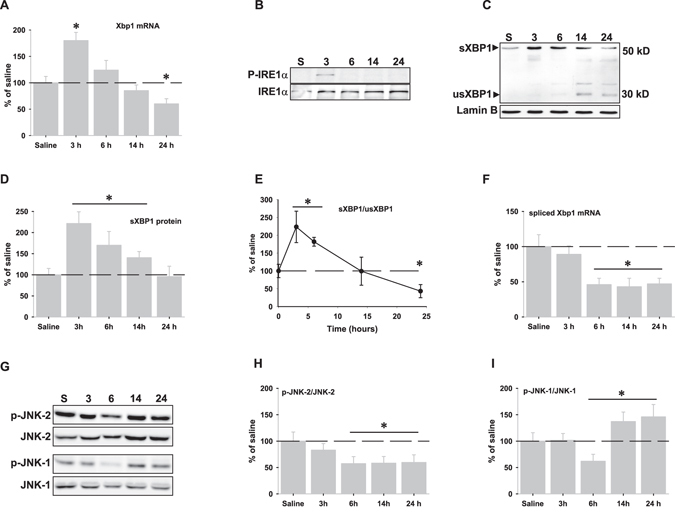
IRE1α pathway is activated following LPS injection. (**A**) mRNA expression of XBP1 analyzed by RT-real time PCR at different times after LPS injection. (**B**) Representative western blot of the phospho-IRE1α (P-IRE1α) and IRE1α proteins in the cytosolic fractions at different times after LPS injection. Note the short time expression. (**C**) XBP1 protein expression in the nuclear fractions after LPS injection. The band around 50 kDa corresponds to the spliced active transcription factor (sXBP1), while the other one, around 30 kDa corresponds to the un-spliced (usXBP1) protein. Note the time-dependent increase of the usXBP1 protein. (**D**) Optical density analysis of western blots corresponding to the sXBP1 protein. (**E**) Graphical representation of the sXBP1/usXBP1 protein ratio. (**F**) mRNA expression of spliced XBP1 analyzed by RT-real time PCR. (**G**) Representative western blots of phospho-JNK2 and total JNK2 and phospho-JNK1 and total JNK1 in the cytosolic fractions at different times after LPS injection. Note both JNK1 and 2 inactivation at six hours after LPS injection. (**H** and **I**) Graphical representation of the phospho-JNK1/JNK1 and phospho-JNK2/JNK2 ratios, respectively determined by optical density analysis. Data are presented as mean ± SD (n = 4) of percentage of variation relative to controls. *p < 0.05, significant differences compared with control (saline-injected) animals.

As shown in Fig. 3A, LPS injection produced mRNA up-regulation of the *Xbp1* gene and the phosphorylation of IRE1α (Fig. 3B), indicating that the IRE1α pathway was early activated after LPS injection. The Xbp1 mRNA is spliced by the nuclease activity of the phospho-IRE1α, leading after translation, to the active transcription factor XBP1 (sXBP1). In this regard, the amount of sXBP1 increased significantly in the nuclear fractions at 3 hours, decreasing afterwards in a time-dependent manner (Fig. 3C and D). By contrast, the unspliced XBP1 protein (usXBP1), which is produced by translation of the Xbp1 mRNA (not spliced), increased in an opposite manner (Fig. 3C). This resulted in a time-dependent switch in the sXBP1/usXBP1 protein ratio (Fig. 3E). Because IRE1α activation was observed for no longer than 3 hours, we further investigated whether the time-dependent reduction in the sXBP1 protein could be explained by a reduction in the amount of spliced Xbp1 mRNA. In this regard, data demonstrated a time-dependent and significant decrease in the content of spliced Xbp1 mRNA that paralleled the sXBP1protein decrease (Fig. 3F).

On the other hand, the IRE1α arm activates the c-Jun N-terminal kinase (JNK) pathway through the formation of an IRE1α-TRAF2-ASK1 complex. As shown in Fig. 3G–H, LPS injection decreased phosphorylation of both JNK isoforms at 6 hours, supporting JNK inactivation. However, from 6 to 24 hours the phospho-JNK-2/JNK-2 ratio remained low, whereas the phospho-JNK-1/JNK-1 ratio was significantly increased, suggesting that LPS-induced neuroinflammation might affect differentially both JNK isoforms.

### ATF6α pathway

**Figure 4:**
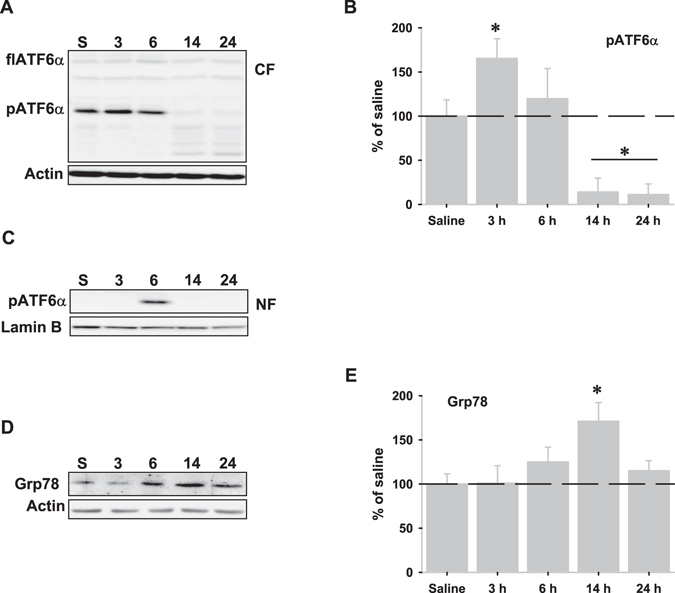
ATF6α pathway is activated following LPS injection. (**A**) Representative western blot of the ATF6α protein in the cytosolic fractions after LPS injection. Note the depletion of the processed ATF6α (pATF6α) but not of the full length ATF6α (flATF6α) at 14 and 24 hours. (**B**) Graphical representation of the pATF6α expression in the cytosolic fractions determined by optical density analysis. (**C**) Representative western blot of the pATF6α in the nuclear fractions. Note the short time expression. (**D**) Representative western blot of Grp78 protein following LPS-injection. (**E**) Graphical representation of Grp78 expression in the cytosolic fractions determined by optical density analysis. Data are presented as mean ± SD (n = 4) of percentage of variation relative to controls. *p < 0.05, significant differences compared with control (saline-injected) animals.

LPS injection also activated the ATF6α arm. While the full-length ATF6α (flATF6α) protein seemed not to be altered by LPS injection, its processing was activated leading to a significant increase of the processed ATF6α (pATF6α) protein in the cytosolic fractions, 3 hours after LPS injection (Fig. 4A and B), and later (6 hours) in the nuclear fractions (Fig. 4C). Accordingly, glucose regulated protein 78 (Grp78), which is mostly under the control of pATF6α^23^ increased from 6 to 14 hours (Fig. 4D and E). Importantly, the ATF6α pathway was briefly activated as revealed by the absence of pATF6α in the nuclear fractions beyond 6 hours, and the fact that it was almost depleted from 14 to 24 hours from the cytosolic fractions (Fig. 4A).

### PERK pathway

**Figure 5:**
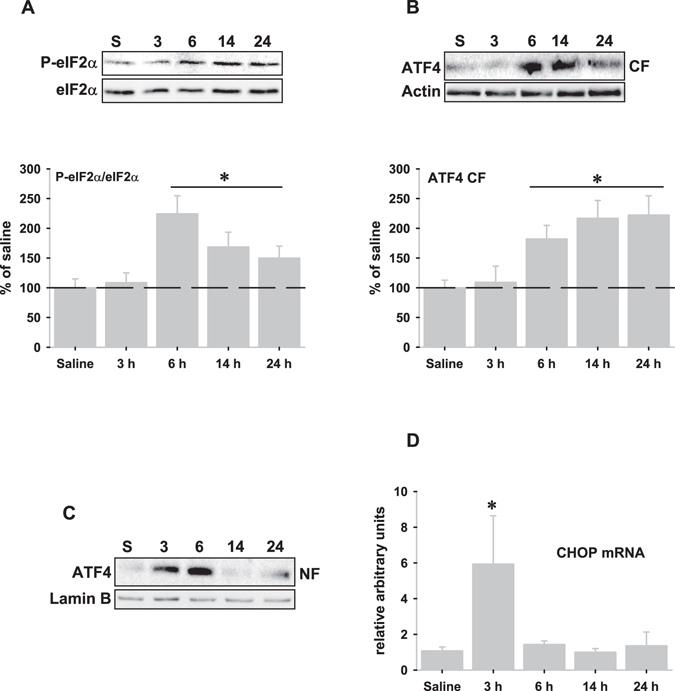
PERK pathway is activated following LPS injection. (**A**) Representative western blot of the phospho-eIF2α (P-eIF2α) and eIF2α proteins expressed in the cytosolic fractions after LPS injection (upper panel), and graphical representation of the P-eIF2α/eIF2α ratio in the cytosolic fractions determined by optical density analysis (lower panel). (**B**) Representative western blot of the ATF4 expression in the cytosolic fractions after LPS-injection (upper panel), and graphical representation of ATF4 expression in the cytosolic fractions determined by optical density analysis (lower panel). (**C**) Representative western blot corresponding to the transcription factor ATF4 in the nuclear fractions of the same animals following LPS-injection. Note the presence of nuclear ATF4 at 3 hours, preceding cytosolic ATF4 accumulation. (**D**) mRNA expression of the pro-apoptotic factor CHOP analyzed by RT-real time PCR after LPS-injection. Note the short time expression of CHOP mRNA. Data are presented as mean ± SD (n = 4) of percentage of variation relative to controls. *p < 0.05, significant differences compared with control (saline-injected) animals.

As shown in Fig. 5A, LPS injection significantly increased the phospho-eukaryotic initiation factor 2α (peIF2α)/eIF2α ratio and the amount of the PERK-related transcription factor ATF4 from 6 to 24 hours (Fig. 5B), indicating PERK activation. However, the presence of ATF4 in the nuclear fractions was evident from 3 to 6 hours, suggesting that LPS-induced nuclear translocation of ATF4 might occur in a PERK-independent manner. Indeed, transcriptional induction of the transcription factor C/EBP homologous protein (CHOP), which is under the control of ATF4, was significantly upregulated 3 hours after LPS injection. However, CHOP protein was not observed at any time (data not shown).

All in all, these data support that LPS injection alters hippocampal ER homeostasis leading to full UPR activation. Moreover, the three pathways showed different time courses of activation/deactivation being the ATF6α the later activated but the earliest attenuated.

### LPS injection down-regulates ERAD markers expression

**Figure 6:**
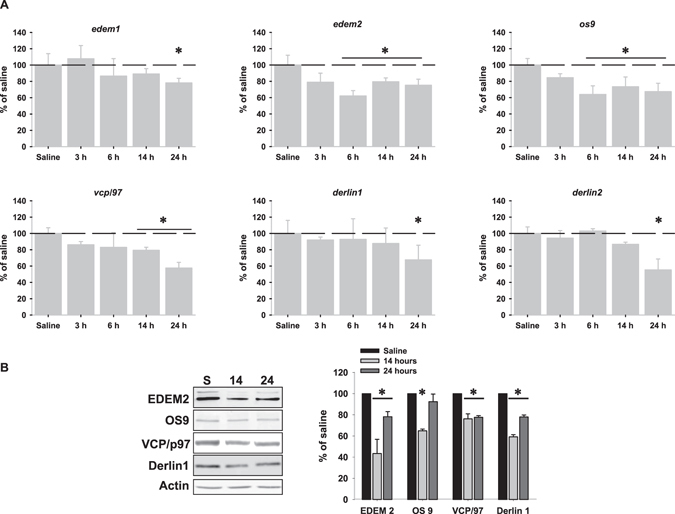
LPS injection down-regulated ERAD markers expression. (**A**) mRNA expression of ERAD markers involved in both recognition (upper panel) and retro-translocation (lower panel) of unfolded and/or malfolded ER proteins analyzed by RT-real time PCR after LPS-injection. Note the significant reduction in the expression of all of the markers analyzed at 24 hours. (**B**) Representative western blot corresponding to selected ERAD proteins of the same animals at 14 and 24 hours after LPS-injection. Data are presented as mean ± SD (n = 4) of percentage of variation relative to controls. *p < 0.05, significant differences compared with control (saline-injected) animals.

ERAD markers are under the control of both pATF6α and sXBP1^23^. To investigate whether nuclear depletion of both pATF6α and sXBP1 could have functional relevance, we evaluated ERAD activation by analysing the expression of several ERAD-associated molecules involved in both recognition and retrotranslocation of misfolded and/or unfolded proteins. Interestingly, despite LPS injection produced UPR activation none of the ERAD markers analyzed was transcriptionally up-regulated. By contrast, the mRNA expression of ERAD markers tended to decrease in time-dependent manner, being all of them significant at 24 hours (Fig. 6A). Importantly, protein analysis also revealed a significant reduction in the amount of some ERAD proteins (Fig. 6B). Taken together, these data demonstrate that LPS injection inactivated ERAD by down-regulating ERAD markers, likely as consequence of the nuclear depletion of both transcription factors pATF6α and sXBP1.

### LPS injection attenuates lactacystin-induced up-regulation of ERAD markers

**Figure 7:**
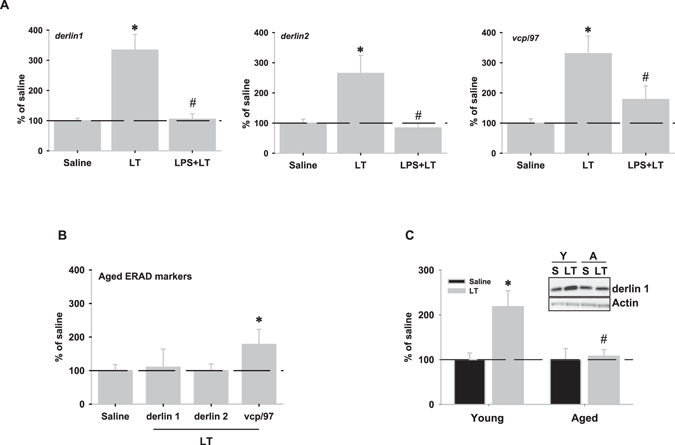
LPS injection attenuates lactacystin-induced up-regulation of ERAD markers. (**A**) mRNA expression of several ERAD markers was analyzed by RT-real time PCR 24 hours after saline (SAL), saline + lactacystin (LT) or LT-injection 24 hours after LPS-injection (LPS+LT). LT-injection induced a significant increase in the mRNA expression of the three ERAD markers, *derlin1*, *derlin2* and *vcp/97*, compared to SAL animals. This mRNA up-regulation was abolished (*derlin1* and *derlin2*) or attenuated (*vcp/97*) in animals that previously received LPS-injection (LPS+LT). (**B**) mRNA expression of ERAD genes in aged rat hippocampus 24 hours following LT-injection. Note that LT-injection just up-regulated mRNA expression of *vcp/97* in aged animals, while the rest remained similar to aged saline-injected rats. (**C**) Representative western blot and quantification by optical density corresponding to the protein Derlin1 in both young and aged rat hippocampus 24 hours after LT-injection. Note the absence of induction in aged rats. Data are presented as mean ± SD (n = 4 young, and n = 3 aged rats) of percentage of variation relative to controls. *p < 0.05, significant differences compared with control (saline-injected) animals. ^#^p < 0.05, significant differences compared with young LT-injected rats.

ERAD activity should be adapted in response to different stressors such as proteasome inhibition to restore proteostasis. Therefore, we tested whether LPS-induced ERAD inactivation could affect ERAD activation induced by proteasomal stress. To address this issue we analyzed the expression of ERAD markers in rats subjected to proteasome inhibition alone (SAL+LT group), and in rats injected with LPS 24 hours before proteasome inhibition (LPS+LT group). In both groups, ERAD expression was analyzed 24 hours after LT injection. As expected, LT injection activated ERAD as revealed by the significant mRNA up-regulation of *derlin1, derlin2* and valosin containing protein (*vcp)/97* (Fig. 7A). However, when proteasome was inhibited 24 hours after LPS injection (LPS+LT), LT-induced upregulation was significantly reduced when compared with LT-injected animals (SAL+LT). Therefore, this data suggests that neuroinflammation could affect ERAD activation under proteasome stress. To test this possibility in a more physiological situation, we induced proteasome inhibition in aged rats, which develop low-grade chronic neuroinflammation^9, 12, 24^, and we analyzed ERAD markers. Importantly, basal expression of ERAD markers was significantly increased in aged rats, suggesting a higher basal ER-stress (Supplementary Fig. S4), and differences between acute versus chronic neuroinflammation. However, and contrary to young rats, lactacystin injection just up-regulated the mRNA expression of *vcp/97* in aged animals, while the other markers remained similar to saline-injected rats (Fig. 7B). These differences were also evident at the protein level as revealed the significant increase of Derlin 1 in young, but no in aged, LT-treated rats (Fig. 7C). These findings strongly support that age-related neuroinflammation might be a relevant factor contributing to defective ERAD activation under proteasome stress.

## Discussion

Here, we provide solid evidence showing that neuroinflammation alters hippocampal proteostasis at different levels. Our findings demonstrate that LPS-induced neuroinflammation produced: (i) autophagy activation; (ii) UPR activation; (iii) ERAD down-regulation; and (iv) ERAD inactivation under proteasomal stress.

It is well established that in both aged hippocampus and neuroinflammation models, the constitutive proteasomes are replaced by the immunoproteasomes^9, 13, 25–27^. We had previously demonstrated that LPS injection induced proteasome to immunoproteasome exchange that is accompanied by a transient reduction of proteasomal proteolytic activity^13^. Here, we show that LPS-induced neuroinflammation activated autophagy at the same time constitutive proteasomes were replaced by the immunoproteasomes. The expression of several macroautophagy-related markers such as LC3-II, Atg7, *bag3* and p62 was altered by LPS injection. Moreover, the master transcription factor TFEB that coordinates autophagy activation and lysosomal biogenesis, increased after LPS injection, and the level of both pro-cathepsin and active cathepsin D were altered. In consequence, we propose that during neuroinflammation, autophagy might be acting as a compensatory mechanism in order to cope with the transient loss of proteolytic activity due to the proteasome to immunoproteasome exchange. These findings reinforce the idea that insufficient proteasomal activity, whatever the reasons, could activate autophagy, and support the functional cooperation between both proteolytic systems^1, 28–30^.

Autophagy is a catabolic process that can be activated in response to a multitude of stimuli through different cellular pathways^22, 31, 32^. In this regard, our data indicated that autophagy activation seemed to be dependent, at least in part, on GSK-3β activity. Interestingly, GSK-3β activity has also been shown to increase autophagic flux in adult hippocampal neural stem cells after insulin withdrawal^33^ and increased GSK-3β activity during ER-stress has been described in the context of neuroinflammation^19^. However, we cannot discard other factors, in addition to GSK-3β activity, that could contribute to LPS-induced autophagy activation. ER-stress markers such as Grp78 up-regulation, sXBP1depletion, PERK/eIF2α phosphorylation or ATF4 stabilization, all of them observed in this study, have been shown to participate in the UPR-induced autophagy activation^30, 34, 35^. Taken toghether, all these data indicate that activation of the axis ER-stress-GSK-3β-autophagy might be relevant during neroinflammation. Surprisingly, we also observed activation of the Akt/mTOR/S6K pathway, a situation that could be compatible with autophagy inhibition^36^. In this case, Akt/mTOR/S6K activation would be modulating excessive autophagy through mTOR activation in order to limit cell death during neuroinflammation as previously suggested^22^. In global, neuroinflammation could modulate autophagy by inducing two opposite pathways in order to get a precise control on autophagy activity. LPS injection also produced a full but faint UPR activation, supporting that neuroinflammation induces ER-stress. Importantly, ER-stress and UPR are related to the induction of proinflammatory cytokines. In particular, XBP1 is activated by TLR2 and TLR4 receptors in macrophages^17^ and it is involved in TNFα production and regulation of innate immune response^17, 19^. In consequence, LPS injection might activate the IRE-1α-XBP1 axis through TLR4 receptor to stimulate TNFα production. In this regard, expression of TNFα, but not of IL-1β or iNOS, took place from 3 to 6 hours after LPS injection^13^. However, we also observed a weak and short activation of both ATF6α and IRE1α pathways. Interestingly, LPS injection gave rise to a time-dependent accumulation of the usXBP1 protein, which inversely correlated with both decreased sXBP1 and pATF6α depletion from the nuclear fractions. In particular, pATF6α was not longer detected in the nuclear fractions beyond 6 hours and was completely depleted from the cytosolic fractions from 14 to 24 hours, when usXBP1 protein levels were maximal. The cellular content of usXBP1 is tightly regulated^37^ and it has been shown to interact with both sXBP1 and pATF6α, but not with ATF4^38, 39^, sequestering them from the nucleus and increasing proteasomal-dependent degradation of these complexes^37^. All these data strongly suggest the interesting possibility that under *in vivo* neuroinflammation, usXBP1 might be modulating the duration and/or intensity of the UPR. In fact, we did not observe activation of ERAD, but rather its inactivation by down-regulation of ERAD-associated molecules, nor CHOP expression^17^. An important issue will be to understand the reasons leading to usXBP1 accumulation in the setting of neuroinflammation. In this regard, we demonstrated that LPS injection produced both a reduction in the amount of sXbp1 mRNA, which support a lower IRE1α-mediated RNAase activity, and GSK-3β activation, two effects that seem to be causally related since GSK-3β activation inhibits the endonuclease activity of IRE-1α^19^. Thus, it is tempting to speculate that LPS-induced activation of GSK-3β might be involved, somehow, in the inhibition of IRE1α-mediated RNAase activity. However, we cannot discard that other factors such as S-nitrosylation might be affecting the IRE1α-mediated RNAase activity in the context of neuroinflammation^40, 41^.

Present data might be relevant in the context of normal aging for which low-grade chronic neuroinflammation is a hallmark^9^. In this regard, we have previously shown that proteasome inhibition in aged rat hippocampus produced: (i) weak activation and rapid attenuation of both IRE-1α and ATF6α; (ii) usXBP1 accumulation; (iii) GSK-3β activation; and (iv) neurodegeneration^1, 7^. Moreover, here we have demonstrated that ERAD was not properly activated following proteasome inhibition in aged rats. Importantly, all of these age-related alterations were reproduced in young LPS-injected rats, pointing out to neuroinflammation as a negative element disturbing cellular proteostasis and increasing neurodegeneration^13^.

In conclusion, a better understanding of how UPR activation is afected in the context of inflammation could have a crucial role in the comprehension of some diseases such as bowel diseases, neurodegenerative diseases or even aging and might help in developing relevant therapeutic tools for treating theses diseases or, at least, alleviating their symptoms.

## Methods

### Animals

Young (3–4 months old) and aged (24–26 months old) male Wistar rats were provided by the animal care facility of the University of Seville. Animals were maintained at 22 to 24 °C and had free access to food and water. All experiments were approved by the ethical committee of the University of Seville and complied with international animal welfare guidelines.

### Surgery

Young rats (n = 28) and aged rats (n = 3) were processed for surgery and drug injection exactly as previously described^13^. Different groups of animals were established. i) Young rats injected with LPS (Sigma-Aldrich, St Louis MO, USA). LPS was dissolved (5 mg/ml) in a solution of sterilized PBS and 1 μl was injected into both hippocampi. The rats were anesthetized with 400 mg/kg chloral hydrate and positioned in a stereotaxic apparatus (Kopf Instruments, Tujunga, CA). According to Paxinos’ atlas the coordinates were: 3.3 mm posterior, 1.6 mm lateral and 3.2 mm ventral to the bregma and 4.8 mm posterior, 5.5 mm lateral and 6.0 mm ventral to the bregma. The injections were delivered over a period of 2 min, and after each, the needle was left *in situ* for an additional 5 min to avoid reflux along the injection track. After deep anesthesia with sodium pentobarbital animals were sacrificed at 3, 6, 14 and 24 hours after LPS injection and brains were quickly removed. Control animals were processed in a similar way but received 1 μl of sterilized PBS in both hippocampi. ii) Young rats injected with saline + lactacystin (LT) or LPS + lactacystin (LPS+LT). In this case, LT (Sigma-Aldrich, St Louis MO, USA) was dissolved (5 mg/ml) in a solution of sterilized PBS and 1 μl was injected into both hippocampi. For each case, saline or LPS was first administered and 24 hours later, LT was injected through the same drilled hole. Finally, after deep anesthesia with sodium pentobarbital animals were sacrificed 24 hours after the last injection. iii) Aged rats injected with LT. Aged rats were processed similarly as LPS injected rats but the coordinates were: 6.0 mm posterior, ±4.6 mm lateral and 4.6 mm ventral to the bregma as previously shown^7^. After deep anesthesia with sodium pentobarbital, animals were sacrificed 24 hours after LT injection. For all cases both hippocampi were dissected, frozen in liquid N_2_, and stored at −80 °C until use.

### Sample preparation

Hippocampi were homogenized in 700 μL of ice-cold sucrose buffer (0.25 M sucrose, 1 mM EDTA, 10 mM Tris-HCl, pH 7.4), supplemented with a protease inhibitor cocktail (Sigma-Aldrich, St Louis MO, USA). Three hundred μL were separated and subsequently used for RNA isolation (see below). The remaining homogenate (400 μL) was stored at −80 °C until use.

### Subcellular fractionation

The nuclear and the cytosolic fractions were obtained from the 400 μL homogenate by sequential centrifugation steps as previously described^27^. The homogenized sample was first cleaned by centrifugation at 500 g for 10 min, and the supernatant recentrifuged at 600 g for 10 min. Then, the supernatant was further centrifuged at 3000 g for 10 min and the pellet was re-suspended in 300 μL of sucrose buffer with protease inhibitors and centrifuged at 3000 g for 10 min. This step was repeated twice. The final pellet, considered as the nuclear fraction, was resuspended in sucrose buffer with protease inhibitors and stored at −80 °C until use. The cytosolic fraction was obtained by centrifugation of the first 3000 g supernatant at 15000 g for 45 min. The resulting supernatant, considered as the cytosolic fraction, was stored at −80 °C until use. The purity of both cellular fractions was tested by western blot using the nuclear protein antibody Lamina. Protein concentration was determined in each fraction by the Lowry method.

### Western blotting

Immunoblots were done as previously described^1^. Briefly, proteins were loaded on a 12 or 14% polyacrylamide gel for electrophoresis (SDS-PAGE; Bio-Rad, Alcobendas, Spain), and then transferred to a nitrocellulose membrane (Hybond-C Extra; Amersham, Barcelona, Spain). After blocking, membranes were incubated overnight at 4 °C with the following primary antibodies: (i) rabbit polyclonal antibodies against: XBP1 (ab37151) at 1 μg/mL; VCP-97 (ab11433) at 1/2000 dilution and lamin B at 1/1000 dilution from Abcam, Cambridge, UK. Grp78 (#SPA-826) at 1/2000 dilution from Stressgen. EDEM2 (E1782) at 1 μg/mL from Sigma-Aldrich, Madrid, Spain. Phospho-S6K (sc-11759) at 1/1000 dilution and ATF6α (sc-22799) at 1⁄1500 dilution from Santa Cruz Biotechnology, USA. LC3B (#2775); Atg7 (#2631); p62 (#5114); phospho-Akt (#9271); Akt (#9272); phospho-GSK-3β (#9336); phospho-JNK (#9251); JNK (#9252); eIF2α (#9722); ATF4 (#11815); Derlin1 (#8897); mTOR (#2972); phospho-mTOR (#2971) at 1/1000 dilution from Cell Signaling, Danvers, MA, USA. TFEB (NB100–93447) at 1/1000 dilution from Novus, Madrid, Spain. (ii) Mouse or rabbit monoclonal antibodies against IRE1α (#3294); OS9 (#12497); Phospho-eIF2α (#3597) at 1⁄1000 dilution from Cell Signaling. GSK-3β (#05-412) at 1⁄1000 dilution from Millipore. β-catenin (ab32572) and phospho-IRE1α (ab124945) at 1⁄1000 dilution from Abcam, Cambridge, UK. Cathepsin D (AF1C7) at 1⁄2000 dilution from AbFrontier Seoul, Korea. β-actin (A 5316) at 1⁄1000 dilution from Sigma-Aldrich, Madrid, Spain. Then, membranes were incubated with the appropriate secondary antibodies horseradish peroxidase-conjugated from Dako, Denmark at a dilution of 1/10000 and developed using the ECL-plus detection method (Amersham, Barcelona, Spain) and the ImageQuant LAS 4000 MINI GOLD (GE Healthcare Life Sciences, Barcelona, Spain). For quantification, the optical density of individual bands was analyzed using PCBAS 2.08 software (Raytest Inc, Berlin, Germany), and normalized relative to the optical density of β-actin or lamin B.

### RNA extraction and reverse transcription

For PCR analysis, total RNA was extracted using the Tripure Isolation Reagent (Roche, Mannheim, Germany) according to the instructions of the manufacturer. The recovery of RNA was similar in young and aged rats. Reverse transcription was performed using random hexamers primers exactly as previously described^1^.

### Real-time PCR

PCR was performed in an ABI Prism 7000 sequence detector (Applied Biosystems, Madrid, Spain) using cDNA diluted in sterile water as template. The *xbp1, chop, Atf6, grp78, derlin1, derlin2, vcp/p97, edem1, edem2, os9* and the housekeeper genes were amplified using specific Taqman probes supplied by Applied Biosystems, Madrid, Spain. The spliced Xbp1 mRNA was amplified using the following pair of primers (from 5′ to 3′): *sXbp1UP*: GCTTGTGATTGAGAACCAGG and *sXbp1LO*: GGCCTGCACCTGCTGCGGACTC^7^. Threshold cycle (Ct) values were calculated using the software supplied by Applied Biosystems, Madrid, Spain.

### Cell culture

Microglial N13 cells were cultured in RPMI 1640 medium (Sigma-Aldrich, Madrid, Spain), supplemented with 10% heat-inactivated fetal bovine serum, 100 U/ml penicillin, and 100 mg/ml streptomycin (Euroclone, Milano, Italy). Cells were stimulated with 0.5 μg/ml of LPS (L3024 S), from Sigma-Aldrich, Madrid, Spain, for 5 hours in the presence or the absence of 20 μM of the GSK-3β inhibitor VII (361548) from Calbiochem, UK, or 0.5 μg/ml of LPS for 4 hours followed by 1 μM of bafilomycin A1 (CAS 88899-55-2) for 5 more hours. Samples were collected using loading sample buffer and processed for Western blot experiments.

### Statistical analysis

Data were expressed as mean ± SD. For comparison between several groups, we used a multifactor ANOVA, followed by Bonferroni post hoc multiple comparisons tests (Statgraphics plus 3.1) (Warrenton, VA, USA). The significance was set at 95% of confidence. Significant differences are referenced as P < 0.05: *respect to saline-injected animals or, ^#^respect to young animals. The comparison between saline and LT injected aged rats was done by two-tailed t test.

## Electronic supplementary material

Supplementary information
