# Supplementary material for: Neuroinflammation alters cellular proteostasis by producing endoplasmic reticulum stress, autophagy activation and disrupting ERAD activation
Source: Sci Rep. 2017 Aug 14;7:8100. doi: 10.1038/s41598-017-08722-3 (PMC5556015; doi:10.1038/s41598-017-08722-3)
Supplement: Supplementary file 1 — Supplementary information [file 41598_2017_8722_MOESM1_ESM.pdf]

Supplementary Information

Neuroinflammation alters cellular proteostasis by producing endoplasmic reticulum stress, autophagy activation and disrupting ERAD activation

Cristina Pintado, Sandra Macías, Helena Domínguez-Martín, Angélica Castaño and Diego Ruano

Supplementary information Fig S1

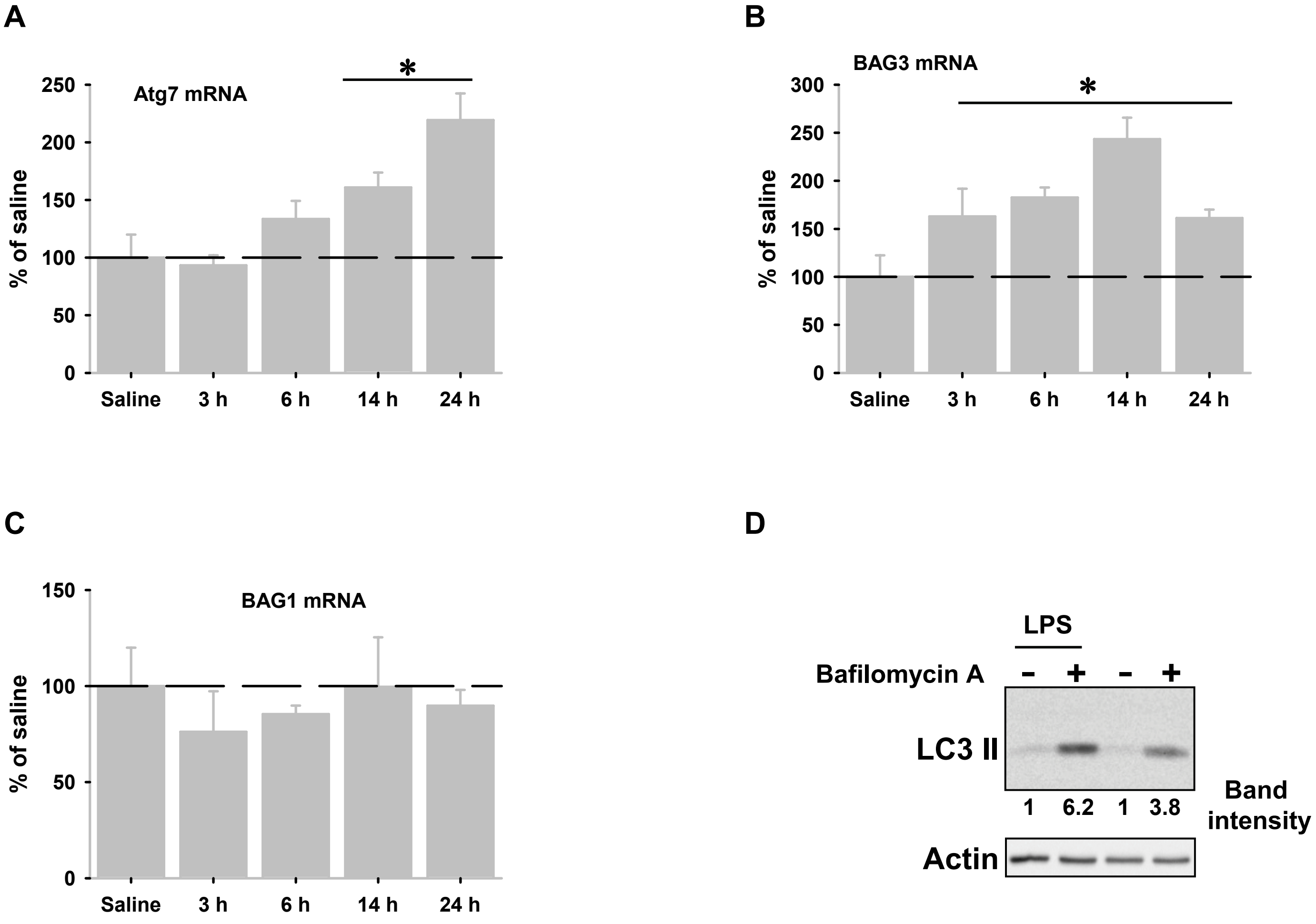

Supplementary information Figure S1. LPS-injection induced transcriptional expression of some autophagy related markers and increased autophagic flux. (A-C) mRNA expression *atg7, bag3 and bag1*, respectively was analyzed by RT-real time PCR in rat hippocampus following LPS-injection. Both *atg7*, involved in autophagy activation, and *bag3*, involved in delivering proteins for autophagic degradation, were significantly up-regulated. By contrast, the mRNA expression of *bag1*, involved in delivering proteins for proteasomal degradation, remained unaltered. (D) Autophagic flux was assessed in N13 cells using bafilomicyn A. Note accumulation of LC3 II, following bafilomicyn A incubation. Importantly, LC3 II band intensity was higher in LPS treated cells than in control cells.

Supplementary information Fig S2

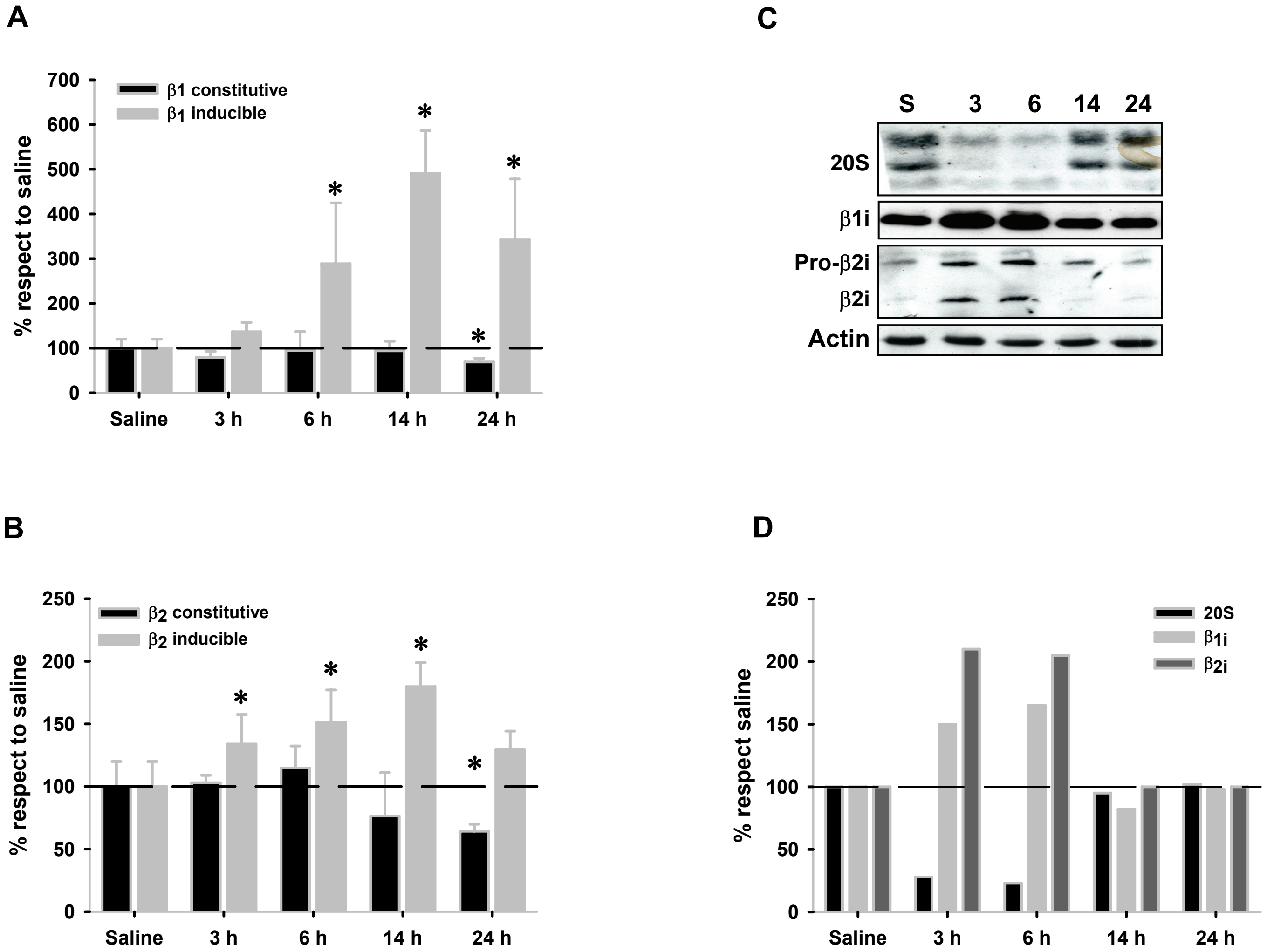

Supplementary information Figure S2. LPS-induced autophagy activation is coincident in time with immunoproteasome expression. (A-B) LPS-injection induced early transcriptional expression of both the β1i and β2i immunoproteasome subunits, whereas that of the constitutive β1 and β2 subunits remained unaltered or even down-regulated. (C) At the protein level, both immunoproteasome subunits β1i and β2i showed a marked expression during the first 6 hours after LPS injection, at the same time several 20S proteasome subunits decreased (D) Graphical representation of data from optical density quantification of western blots corresponding to β1i, β2i and 20S proteasome subunits. Data are presented as mean $\pm$ SD (n= 4) of percentage of variation relative to controls. * $p < 0.05$, significant differences compared with control (saline-injected) animals.

Supplementary information Fig S3

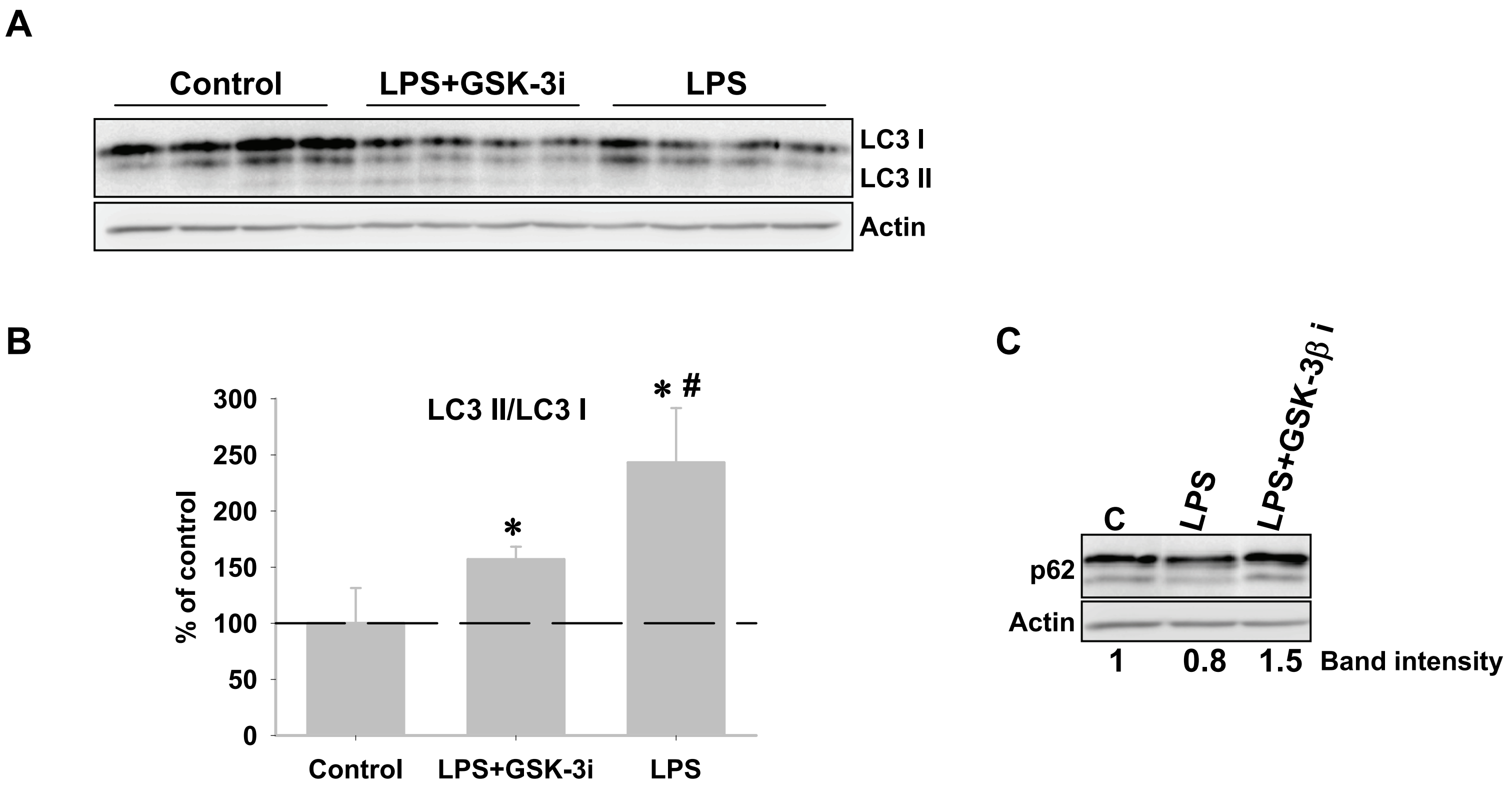

Supplementary information Figure S3. GSK-3β inhibition reduced LPS-induced autophagy activation. (A) Microglial N13 cells were stimulated with 0.5μg/ml of LPS for 5 hours in the presence or the absence of 20 μM of the GSK-3β inhibitor VII. Samples were collected and LC3 expression was analyzed by western blot. (B) Wester blot was quantified by optical density and data are represented in the graph. Data are presented as mean $\pm$ SD (n= 4) of percentage of variation relative to control cells. * $p < 0.05$, significant differences compared with control cells. # $p < 0.05$ compared to LPS treated cells. (C). Representative western blot of p62 protein of the same cultures showed in A.

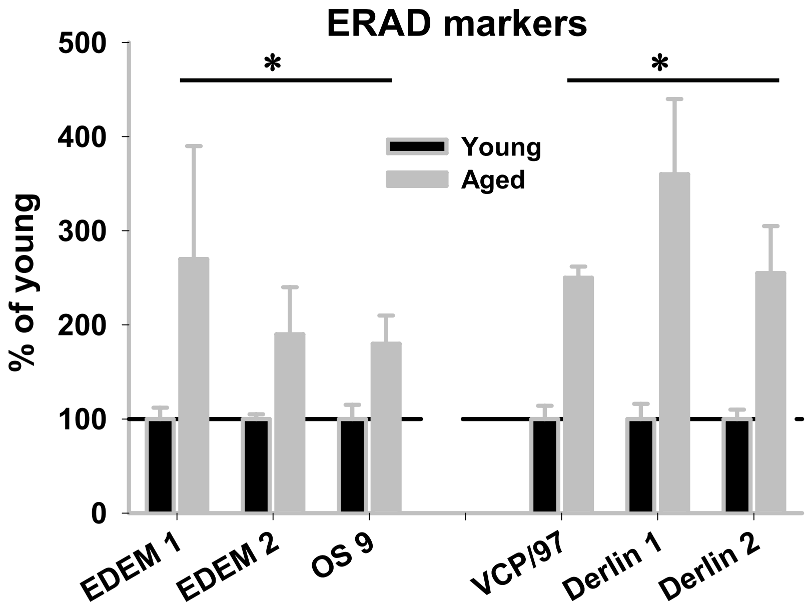
ERAD markers
500
400
300
200
100
0
% of young
*
*
Young
Aged
EDEM 1
EDEM 2
OS 9
VCP/97
Derlin 1
Derlin 2

Supplementary information Figure S4. ERAD markers expression in young and aged rat hippocampus. mRNA expression of several ERAD markers was analyzed by RT-real time PCR in young and aged rat hippocampus. Basal expression of all of the ERAD markers analyzed was significantly increased in aged animals.
